# Breast Metastasis in Esophagus Cancer: Literature Review and Report on a Case

**DOI:** 10.1155/2016/8121493

**Published:** 2016-05-31

**Authors:** Abdulaziz Ghibour, Osama Shaheen

**Affiliations:** Department of General Surgery, Al-Mouwasat University Hospital, Faculty of Medicine, Damascus University, 5371 Damascus, Syria

## Abstract

Esophagus cancer metastases often involve locoregional lymph nodes, lung, bone, liver, and brain. Metastatic involvement of the breast from esophagus cancer is uncommon, but if it happened, it usually presents as a part of multiple organ distal metastases. Here we report a case of the largest metastatic esophagus cancer of the breast and the chest wall, and we review the similar reported cases.

## 1. Introduction

Esophagus cancer metastases to unexpected sites usually happen when there are widespread metastases to other organs [1], and breast is considered one of the unexpected sites for esophageal cancer metastasis. Here, in order to understand more about this rare phenomenon, we conducted a literature review using Medline, PubMed, and Google Scholar on all the cases that described breast metastasis from esophagus cancer. We report as well a remarkable case with the largest breast metastasis from esophagus cancer.

## 2. Case Report

**Figure 1:**
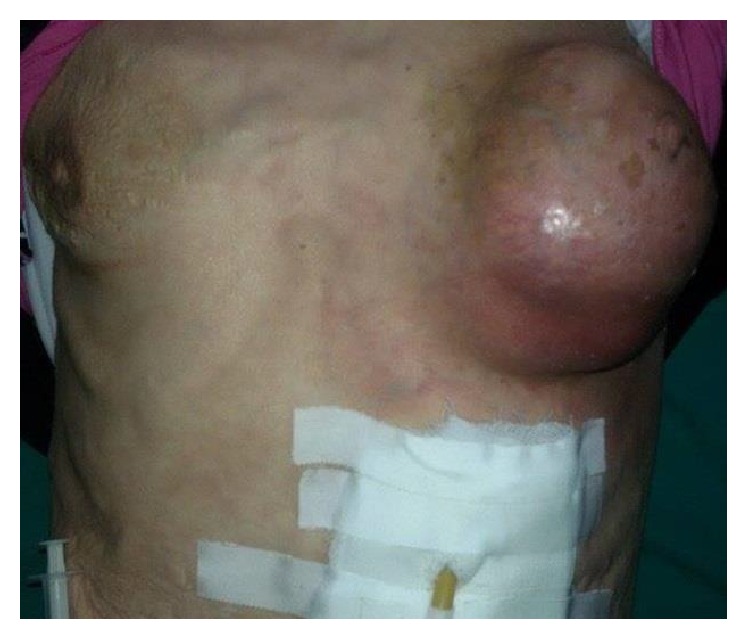
Left breast mass with gastrostomy.

A 57-year-old woman presented to our clinic with a painful left breast mass (Figure 1). The mass started to appear six months previously and gradually increased in size to become painful and tense, but without discharge from the nipple.

**Figure 2:**
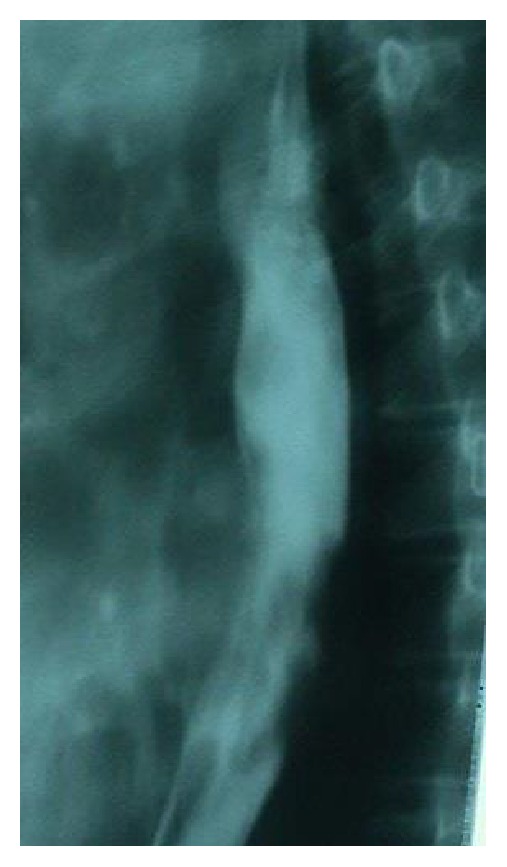
Esophagogram showing signs of mid-lower esophagus mass.

Her past medical history revealed she had been diagnosed with esophagus carcinoma one year ago (Figure 2), but she had refused any kind of treatment back then. Review of her other symptoms showed that the dysphagia associating the esophagus cancer had increased gradually during the last year until it became impossible for her to swallow any solid food during the last month, and she had lost 25 kg during the previous four months.

The patient was severely malnourished, her BMI was 14 kg/m^2^, and her vital signs were as follows: Bp: 100/55 mm/Hg; pulse: 130 beats per minute; temperature: 99.5 F; RR: 22 breath per minute; she was alert and oriented but looked tired. The examination showed a fixed 9 × 10 × 7 cm painful hard mass involving the left breast. The skin over the mass was red but not hot and the rest of the examination including the lymphatic system was unremarkable except a noticeable wheezing in the right chest.

**Figure 3:**
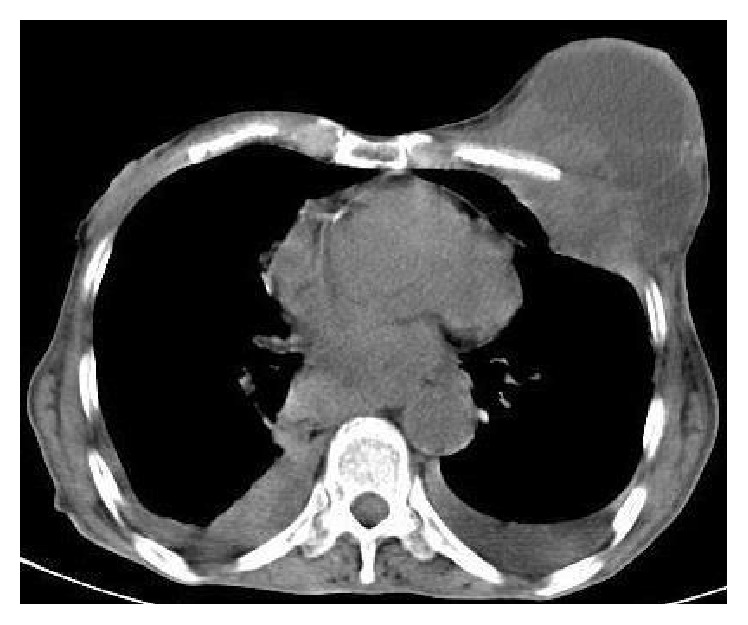
Oral and intravenous contrast-enhanced CT scan of the chest revealed bilateral pleural effusions with breast mass involving the left chest muscles and pleura destroying the rips.

The blood tests were normal except for a decrease in TP and ALB and a mild decrease in calcium. The head, chest, and abdominal CT scan showed a 4 × 6 × 7 cm lobulated mass involving the lower third of the esophagus accompanied with a large (10 × 9.5 × 8 cm) lobulated (with necrotic component) mass involving the left breast, the left chest muscles, and the pleura; this mass is compressing the anterior face of the lung and destroying the accompanied ribs (Figure 3); the CT scan showed as well left and right pleural effusions without any other obvious metastasis.

**Figure 4:**
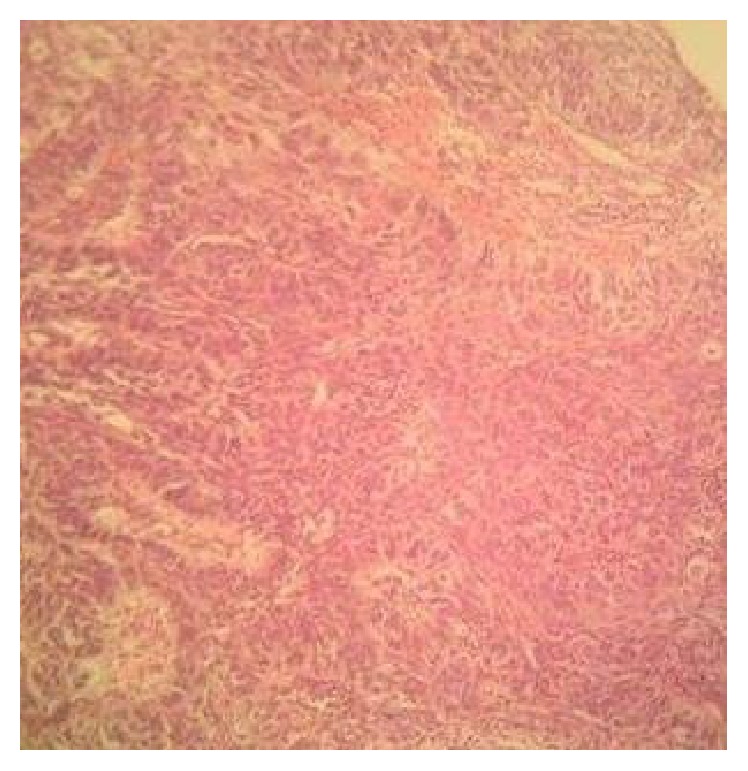
Histological view of the breast mass showing typical structure squamous cell carcinoma (hematoxylin and eosin, ×100).

**Figure 5:**
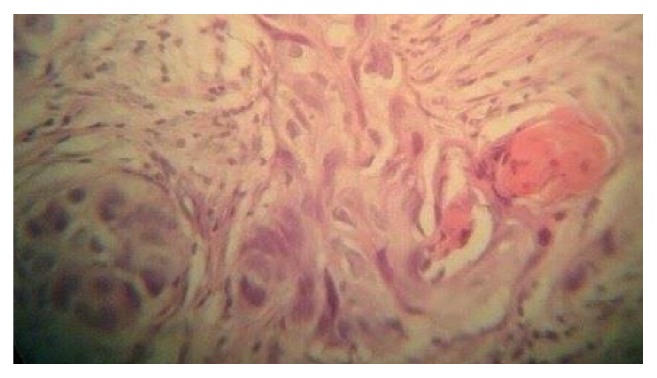
Histological view of the breast mass showing typical structure squamous cell carcinoma (hematoxylin and eosin, ×100).

Review of her medical history revealed moderately differentiated squamous cell carcinoma of the mid-lower third of the esophagus. The breast biopsy showed solid cords, sheets, and lobules of pleomorphic malignant epithelial cells with occasional bizarre, hyperchromic nuclei with occasional keratin pearls compatible with poorly differentiated squamous cell carcinoma (Figures 4 and 5). The pleural effusion examination was negative for malignancy.

Feeding tube gastrostomy was done to the patient; however, she passed away two months later.

## 3. Discussion

In this study we did a literature review to the cases that described metastatic breast disease from esophagus cancer; we will report the clinical, radiological, and pathological features of the breast metastasis and we will review the diagnostic and the treatment options.

Only six cases were found in literature; besides our case the cases were 6 females and one male summarized in Table 1; the tumors were located in the middle and/or lower esophagus in 6 cases (squamous cell carcinoma (SSC) in five cases and adenocarcinoma in one case); only one case was reported as SCC in the upper middle part. These metastases were presented at variable times after the diagnosis of the original tumor (2 to 24 months); only one case reported the metastasis as the first sign of the esophagus cancer [2].

The physical examination of the breast metastasis demonstrated masses indistinguishable from primary mammary carcinoma, although they were often circumscribed and were described as painful in 3 cases. The metastasis size ranges from 2 to 5 cm but it was remarkably larger in our case and reached 10 cm to completely involve the left breast, the left chest muscles, and the pleura. The metastases were located in the left breast in 5 cases and in the right breast in 2 cases.

The mammography was done in 5 cases and was negative for microcalcification. Core-needle or excisional biopsies were used for pathological diagnoses since immunohistochemistry and the presence of an in situ component play an important role in differentiating between primary and metastatic tumors [6]. Immunohistochemistry was always negative for ER, PR, and Her2 neu. In this review breast metastasis was the only recurrence in two cases, and acceptable results without signs of recurrence were mentioned up to six months after surgical resection of the breast metastasis. Only one case reported successful breast and brain metastasis resection outcome 11 years later. In our case, an autopsy was not done to the patient and she refused the treatment from the beginning; nevertheless, the large breast metastasis that involved the chest muscles and the pleura was the only detectable metastasis on CT scan. Three cases out of seven mentioned the presence of multiple untreatable metastasis during management or diagnosis.

We found that many case reports and some experimental studies were published trying to emphasize the complexity of esophagus cancer metastases patterns; these studies found that the complex anatomical pathway of the esophagus lymphatic network can elucidate the possibility of the random distribution of the metastases in esophagus cancer [8]. Despite the high frequency of distant metastasis to the lung, bone, liver, and brain observed in esophagus carcinoma, only a few cases were reported as being related to metastatic breast disease.

The studies showed that the lymphatics originating from the thoracic esophagus frequently drain into the thoracic duct directly without intervening lymph nodes [8, 9]; since the thoracic duct has multiple collateral branches with the intercostal vessels, it can reach the internal mammary chain and may spread to the breast.

In general, treatment should be directed to the primary malignancy; usually the prognosis is poor and surgical resection of breast metastasis suffices only if metastases in other sites are not present or being controlled [5].

In conclusion breast metastasis in esophagus cancer is an extremely rare disease; the diagnosis should be highly suspected in any breast mass with a previous history of esophagus cancer, especially in the presence of multiple metastases. When the breast mass is the only reported metastasis, the case reports revealed that the surgical excision of the metastasis showed an acceptable result on the short-time follow-up; however, more studies are needed for correct treatment choices. We reported the largest breast metastasis in esophagus cancer that involved the left chest muscle and the pleura, without any other obvious radiological metastases. The possibility of this phenomenon may elucidate part of the anatomical network pathway of esophagus cancer.

## Figures and Tables

**Table 1:** Breast metastasis from esophagus cancer—cases characteristics.

Case	Study	Patient sex and age	Tumor location	Tumor pathology	Esophageal cancer treatment	The breast metastasis	Interval between tumor and metastasis	Metastasis management and outcome
1	Nielsen et al., 1981 [2].	84 y/o female	Middle esophagus	Squamous cell carcinoma	No management available	5 cm central mass located in the right breast	Three months after diagnosis	Breast metastasis detected at autopsy

2	Miyoshi et al., 1999 [3].	44 y/o male	Upper middle esophagus	Squamous cell carcinoma	Radiotherapy due to metastasis	Painful mobile hard mass beneath the left nipple	Two months after diagnosis	The patient died 2 months later Autopsy: lung, liver, diaphragm, peritoneum, and spine metastasis

3	Shiraishi et al., 2001 [4].	57 y/o female	Middle esophagus	Squamous cell carcinoma	Esophagectomy Radiotherapy	2.5 cm × 2.6 cm mobile painless hard mass in the upper outer quadrant of the left breast	Two years after surgery	The patient had modified radical mastectomy; she was alive 6 months later

4	Santeufemia et al., 2006 [5].	51 y/o male	Middle esophagus	Squamous cell carcinoma	Esophagectomy Chemotherapy	3 cm × 3 cm hard mobile nodule in the upper lateral quadrant of the left breast	Four months after surgery	Surgical resection of breast and brain relapse. Successful outcome 11 years later

5	Norooz et al., 2009 [6].	35 y/o female	Middle lower esophagus	Squamous cell carcinoma	Esophagectomy Chemotherapy Radiotherapy	4 cm × 4.5 cm mobile, painful, hard mass just below the right nipple	Metastatic breast lesion was the first sign of the esophagus cancer	Resection of the breast mass The patient has acceptable health condition 6 months after the treatment with no sign of recurrence

6	Jena et al., 2014 [7].	32 y/o male	Lower esophagus	Adenocarcinoma	Esophagogastrectomy Chemotherapy	2 cm × 2 cm mobile hard lump in the upper outer quadrant of left breast	Two years after surgery	No management available; the patient died later

7	Our study 2016	57 y/o female	Middle lower esophagus	Squamous cell carcinoma	No management available	10 cm × 9.5 cm painful hard mass involving the left breast	One year after diagnosis	The patient died 2 months later
